# Examining Associations Between Psychopathic Traits and Executive Functions in Incarcerated Violent Offenders

**DOI:** 10.3389/fpsyt.2018.00310

**Published:** 2018-07-11

**Authors:** Carl Delfin, Peter Andiné, Björn Hofvander, Eva Billstedt, Märta Wallinius

**Affiliations:** ^1^Department of Psychiatry and Neurochemistry, Centre for Ethics, Law and Mental Health, Institute of Neuroscience and Physiology, Sahlgrenska Academy, University of Gothenburg, Gothenburg, Sweden; ^2^Research and Development Unit, Regional Forensic Psychiatric Clinic, Växjö, Sweden; ^3^Forensic Psychiatric Clinic, Sahlgrenska University Hospital, Gothenburg, Sweden; ^4^Department of Forensic Psychiatry, National Board of Forensic Medicine, Gothenburg, Sweden; ^5^Department of Clinical Sciences Lund, Child, and Adolescent Psychiatry, Faculty of Medicine, Lund University, Lund, Sweden; ^6^Gillberg Neuropsychiatry Centre, Institute of Neuroscience and Physiology, University of Gothenburg, Gothenburg, Sweden

**Keywords:** psychopathy, executive functions, neuropsychological tests, offenders, violence, crime, prison

## Abstract

Executive functions (EFs) are essential in almost all aspects of daily life and have been robustly related to antisocial behavior. However, the relationship between psychopathy and EFs has remained equivocal. Research investigating lower-level trait dimensions of psychopathy using standardized EF measures could be beneficial in addressing this issue. In this study, we examined associations between four EFs and four dimensions of psychopathic traits (interpersonal, affective, lifestyle, antisocial) using zero-order correlation and a combination of classical and Bayesian statistical methods. Two hundred and fourteen incarcerated male violent offenders were assessed with the Psychopathy Checklist-Revised and completed tests of cognitive flexibility, spatial working memory, response inhibition, and planning and problem-solving using the Cambridge Neuropsychological Test Automated Battery. Lifestyle psychopathic traits were significantly associated with reduced initial thinking time in a planning and problem-solving task, with a Bayes factor indicating substantial evidence for the observed correlation, and antisocial psychopathic traits showed a significant association with reduced initial thinking time in the same task, although the Bayes factor indicated only anecdotal evidence. Significant associations were also found between affective and antisocial psychopathic traits and less efficient strategic thinking in a spatial working memory task, and between affective, lifestyle and antisocial psychopathic traits and fewer problems solved in a planning and problem-solving task, although these findings were not corroborated by the Bayesian analysis. While the observed effects ranged between small and medium, our study suggests that reduced initial thinking times in planning and problem-solving is robustly associated with higher degrees of lifestyle and antisocial psychopathic traits.

## Introduction

Executive functions (EFs) are essential in almost all aspects of daily life. Described as a set of separate but related top-down cognitive processes that come into play whenever automatic behaviors are insufficient or inappropriate, EFs govern self-regulated and goal-directed behavior. It is generally agreed that there are three core EFs—cognitive flexibility, working memory, and inhibition—from which higher order EFs such as planning and problem-solving are built (1, 2). Since EFs affect our daily life, impaired EFs are associated with a wide range of psychopathology, including behavioral disinhibition, which in turn is associated with several externalizing behaviors (3, 4). In correctional settings, behavioral disinhibition has been related to disadvantageous treatment engagement in offenders (5), and a recent longitudinal study found that impaired EFs seem to predispose to recidivism in adolescents with conduct disorder (6). The importance of EFs in correctional settings is further emphasized by meta-analyses showing a robust association between EF deficits and antisocial behavior (7, 8). Antisocial behavior is a heterogenous construct, however, and despite large overlaps in observed behaviors, different forms of antisocial behavior have different underlying etiologies (9). For instance, offenders with high levels of psychopathic traits are more violent, more prone to recidivism and, unfortunately, more resistant to treatment than offenders with low levels of psychopathic traits (10–15). Yet, despite the robust association between EF deficits and antisocial behavior, the role of EFs in psychopathy remains inconclusive. Several studies contrasting individuals with low and high degrees of psychopathic traits have failed to find differences in various EFs (16–18). Other studies suggest that EF deficits are similar in antisocial individuals with and without psychopathy (19, 20), and thus related to general antisocial behavior rather than to psychopathy. As an example of previous inconclusive results, consider the role of response inhibition. Munro et al. (21) found that although offenders committed more commission errors in a Go/NoGo task than healthy controls (indicative of impaired response inhibition) the number of errors were unrelated to the offenders' levels of psychopathic traits. On the other hand, Krakowski et al. (22) found that offenders with a high degree of psychopathic traits committed more commission errors in a Go/NoGo task compared to healthy controls with a low degree of psychopathic traits, suggesting impaired response inhibition in psychopathic individuals. Further complicating matters, (23) compared three groups of offenders with antisocial personality disorder and concurrent low, medium and high degrees of psychopathic traits and one group of healthy controls and found that it was the group with antisocial personality disorder and a *medium* level of psychopathic traits that showed the greatest response inhibition impairments in a Go/NoGo task. Results have been mixed for other EFs as well. For instance, one study found no association between set-shifting ability and degree of psychopathic traits in a sample of offenders (23), while subsequent studies have observed impaired set-shifting in individuals with a high degree of psychopathic traits when contrasted with individuals with low degrees of psychopathic traits (22, 24). The role of working memory in relation to psychopathic traits has been less explored, but there have been studies suggesting a negative association between working memory and impulsive-antisocial traits (25) as well as a positive association between working memory and interpersonal traits (26). Planning and problem-solving is, like working memory, relatively unexplored. One study found that initial thinking times in a planning and problem-solving task were negatively associated with both interpersonal-affective and impulsive-antisocial traits (27), while one study observed a negative correlation between the impulsive-antisocial traits and a planning and problem-solving task (28).

The pattern of inconclusive results may perhaps primarily be attributed to two issues. First, EFs have been measured differently across studies, possibly due to the broad nature of EFs making exact definition and operationalization inherently difficult [e.g., (29)]. The use of standardized test batteries may alleviate some discrepancy, but there are several such batteries available, each with its own advantages and disadvantages, and there is no general consensus on which to choose (3). Second, divergent operationalizations of the psychopathy construct, both regarding whether the construct is best viewed as categorical or dimensional as well as how it should be measured (i.e., which instrument best captures the psychopathy construct) has likely contributed to inconclusive findings [e.g., (30)]. It has been suggested that comparing groups of individuals based on specific cutoffs for psychopathic traits may not be optimal (31–33), and that adopting a dimensional approach while parsing the psychopathy construct into separate underlying traits along a continuum instead facilitates research of more intricate relationships between specific traits and functions (34). Thus, a dimensional approach may be advantageous when investigating EFs in relation to psychopathy.

Common operationalizations of psychopathy can be seen in the use of measures such as the Self-Report Psychopathy scale, currently at its fourth edition [SRP-4; (35)], the Levenson Self-Report Psychopathy Scale [LSRP; (36)], the Psychopathic Personality Inventory, most recently the revised version [PPI-R; (37)], and the Triarchic Psychopathy Measure [TriPM; (38)]. Still, at least in clinical settings, the most widely used instrument to assess psychopathic traits is the Psychopathy Checklist-Revised [PCL-R; (34, 39)]. The PCL-R consists of 20 items rated on a three-point scale, with a maximum score of 40. Typically, individuals scoring ≥ 30 (or similar cutoffs) are categorized as psychopaths. Research has identified a two-factor (40), a three-factor (41, 42), and a two-factor, four-facet structure of the PCL-R (34). In the two-factor model, factor 1 encompasses interpersonal and affective features such as superficial charm, callousness and lack of empathy while factor 2 entails impulsive and criminal behaviors including irresponsibility, juvenile delinquency, and criminal versatility. In contrast, the three-factor model omits almost all of the items associated with criminality, implying that criminality is not a core construct *in* psychopathy but rather a consequence *of* psychopathy. In this model, factor 1 entails an arrogant and deceitful interpersonal style, factor 2 represents a deficient affective experience, and factor 3 represents an impulsive and irresponsible behavioral style. In the two-factor, four-facet model, the two original factors are further parsed into four underlying facets. These facets, separate but moderately correlated, represent interpersonal (e.g., superficial charm, manipulative lying), affective (e.g., callousness, shallow affect), impulsive lifestyle (e.g., need for stimulation, impulsiveness), and antisocial (e.g., juvenile delinquency, criminal versatility) psychopathic traits. A two-factor approach appears most common in research on psychopathic traits and EFs in incarcerated offenders. Several studies have found support for impulsive-antisocial traits being related to reduced EFs, while interpersonal-affective traits have been related to normal or even superior EFs (28, 43–45), although results continue to be inconclusive (46, 47). In the few studies that have investigated EFs in relation to the PCL-R four-facet structure, one found a positive association between working memory and interpersonal facet scores (26), and one has suggested the antisocial facet as related to poor response inhibition, while the affective facet has been related to better response inhibition (48). A more recent study used a global measure of EFs and found that the independent effects of the affective and antisocial facet were related to worse EFs, although only the affective facet remained significant when unique effects were examined (28).

To summarize, EFs have been associated with a wide range of antisocial behaviors, including disadvantageous treatment engagement and recidivism. As mentioned, offenders with a high degree of psychopathic traits display higher rates of violence, are more prone to recidivism, and more resistant to treatment than offenders with low levels of psychopathic traits, yet the role of EFs in relation to psychopathic traits remains uncertain. To address these issues, the present study investigated associations between psychopathic traits and four different EFs in a clinically well-described sample of incarcerated male violent offenders. More specifically, using open statistical analysis (49) with a combination of classical and Bayesian statistical methods, we examined zero-order correlations among four psychopathic traits (interpersonal, affective, lifestyle, antisocial) and measures from three core EFs (cognitive flexibility, working memory, response inhibition) and one higher order EF (planning and problem-solving).

## Materials and methods

### Participants

Participants (*N* = 214) were male violent offenders recruited from the Development of Aggressive Antisocial Behavior Study (DAABS). The DAABS recruited young adult male offenders (aged 18–25 years at inclusion) who were convicted of hands-on violent (including sexual) offenses and imprisoned in one out of nine prisons in the western region of the Swedish Prison and Probation Service between March 2010 and July 2012, with a participation rate of 71%. All assessments were based on file reviews, structured clinical interviews, self-report, observations, and neuropsychological testing. Interviews, observations, and neuropsychological testing were administered during a full day by a clinical psychologist with special training in the methods used. Detailed descriptions of the cohort are provided in previous publications (50–52). In the total DAABS cohort (*N* = 270), 54 offenders did not participate in or complete all the neuropsychological assessments used in this study, and psychopathy ratings were unavailable for two offenders. Thus, the current study sample consisted of 214 male offenders, aged 18–25 at the time of inclusion (*M* = 21.94, *SD* = 1.87).

### Measures

#### Psychopathic traits

Psychopathic traits were measured using the PCL-R (39), which consists of 20 items rated on a three-point scale (0 = does not apply, 1 = may apply or applies in some respects, 2 = does apply). We adopted the four-facet structure of the PCL-R, in which possible scores for facets 1 (interpersonal traits) and 2 (affective traits) ranges from 0 to 8, and possible scores for facets 3 (lifestyle traits) and 4 (antisocial traits) ranges from 0 to 10. The offenders were rated by an experienced and for the task specifically trained psychologist based on all information available from interviews, observations, and files. Training sessions with consensus ratings on participants, led by an experienced PCL-R assessor, were performed to ensure inter-rater reliability. The mean PCL-R total score in the study sample was 17.52 (*SD* = 7.05). Internal consistency was good, with Cronbach's alpha (α) = 0.85 for the total score being slightly above pooled estimates from the PCL-R Technical Manual (53). Cronbach's α was 0.65, 0.81, 0.78, and 0.77 for the interpersonal, affective and lifestyle facets, respectively, indicating lower but adequate internal consistency. The mean corrected item-total correlation for the total score was 0.47, with 0.56, 0.70, 0.64, and 0.61 interpersonal, affective, lifestyle, and antisocial facets, respectively, also in line with the PCL-R Technical Manual. Note that item *N* ranged from 205 to 214 for the total score, and 211 to 214 for the facets.

#### Executive functions

Four subtests of the Cambridge Neuropsychological Test Automated Battery (CANTAB; Cambridge Cognition Ltd., Cambridge, UK) were used to assess EFs: Intra/Extra Dimensional Shift (IED), Spatial Working Memory (SWM), Stop-Signal Task (SST), and Stockings of Cambridge (SOC). The choice of CANTAB and the subtests included in the current study were due to the primary focus on neurodevelopmental disorders (e.g., ADHD) in the DAABS study. CANTAB has been used extensively and successfully in studies on ADHD [see e.g., (54)], but also in research on antisocial and violent behavior (55, 56) and psychopathy (23).

#### Cognitive flexibility

The IED, similar to the Wisconsin Card Sorting Test (57), assesses cognitive flexibility (also known as set-shifting). Two different types of visual stimuli (shapes and lines) are presented in four possible locations on the screen, and participants are required to select one of two shapes, learning from trial and error which one was correct. As the test progresses, the correct shape switches, distracting lines are added, and attention must be shifted to the previously irrelevant lines while ignoring the shapes. Participants progresses through nine stages of increasing difficulty by reaching a certain criterion at each stage. Measures used in the current study were the amount of stages completed and the number of errors made.

#### Working memory

The SWM is a self-ordered searching task assessing spatial working memory ability. Participants search for tokens hidden inside boxes, with the number of boxes gradually increasing from two to eight as the task progresses. Measures used in the current study were number of errors and strategy score. The strategy score, ranging from 0 to 40, is a measure of optimal strategy (58) with higher scores indicating less efficient strategy.

#### Inhibition

The SST was used to assess response inhibition (59). Participants are shown an arrow stimulus pointing either left or right and are instructed to press the corresponding left or right-hand button, but also to withhold their response if they hear an auditory signal. The SST uses a staircase design for the stop-signal delay so that the task adapts to the performance of the participant, resulting in a 50% success rate for inhibition. Measures used were the stop-signal reaction time, measuring the average time (measured in milliseconds but converted to seconds) at which the participant is able to successfully inhibit the prepotent motor response, and the mean correct response time (measured in milliseconds but converted to seconds).

#### Planning and problem-solving

The SOC is a computerized version of the Tower of London task (60). Participants are instructed to move colored balls at the bottom half of the screen to match the arrangement of similar balls on the top half of the screen. Each problem has a specific minimum number of moves required to match the arrangement (from two to five), and participants are encouraged not to begin until they feel confident that they could solve the entire problem. The measures used in the current study were mean initial thinking time (measured in milliseconds but converted to seconds) before attempting to solve a five-move problem, and the number of problems solved in the minimum number of moves, with possible scores ranging from 0 to 12.

### Data analytic strategy

Data preparation and statistical analysis was conducted using R (61) with all R code publicly available at the corresponding authors' GitHub page (https://github.com/carldelfin/EF-psychopathy). Zero-order Pearson product-moment correlation coefficients (Pearson's *r*) and corresponding *p*-values were calculated using the R package psych (62), with statistical significance set to *p* < 0.05. Bayesian analysis was then conducted to corroborate findings from the classical hypothesis testing, using the R package BayesMed (63) with a default Jeffreys–Zellner–Siow prior set-up (64). Posterior distributions were obtained from Markov chain Monte Carlo sampling (65), using 20,000 iterations after an initial 2,000 burn-in iterations were discarded. We report the posterior probability, which is the estimated probability of the observed correlations given the data, along with Bayes factors (BFs), which is a weighted average likelihood ratio with values > 1 indicating a greater likelihood of the data occurring under the alternative hypothesis (H_1_) and values < 1 indicating a greater likelihood of the data occurring under H_0_ (64). Among the benefits of BFs is that they allow researchers to quantify evidence *in favor* of the null hypothesis (H_0_) rather than against it, and that they do not require correcting for multiple comparisons. We present BFs <1/3 as indicating substantial to decisive evidence in favor of H_0_, 1/3 < BF < 1 as anecdotal evidence in favor of H_0_, 1 < BF < 3 as anecdotal evidence in favor of H_1_, and BF > 3 as indicating substantial to decisive evidence in favor of H_1_, adapted from cutoffs from (64).

## Ethics

This study was approved by the Research Ethics committee at Lund University (Dnr: 2009/405). All offenders fulfilling inclusion criteria were approached by study site managers and received oral and written information about the study in accordance with the Declaration of Helsinki. All participants in the study gave written informed consent. A compensation of 200 Swedish kronor (approximately 20 USD) was given after participation. The compensation was small enough to not create an incentive that would compromise the free ground for participating in the study and was approved by the ethics committee. Participants who showed signs of severe psychopathology were given the opportunity to be referred to the prison psychiatrist for further assessment and treatment whenever there was such an option.

## Results

An overview of PCL-R facet scores and performance on EF measures is presented in Table 1.

**Table 1:** Descriptive statistics (*N* = 214).

	**Mean ± SD**	**Range**
PCL-R interpersonal facet score	0.9 ± 1.34	0–8
PCL-R affective facet score	3.15 ± 2.26	0–8
PCL-R lifestyle facet score	6.45 ± 2.61	0–10
PCL-R antisocial facet score	6.3 ± 2.88	0–10
IED stages completed	8.1 ± 1.13	1–9
IED errors	26.7 ± 12.5	7–63
SWM errors	23.14 ± 17.24	0–90
SWM strategy score	32.47 ± 5.12	0–47
SST stop-signal RT	0.19 ± 0.08	0.07–0.74
SST mean correct RT	0.48 ± 0.14	0.3–1.27
SOC MITT	6.03 ± 4.78	0–29.38
SOC problems solved	8.3 ± 1.75	4–12

*SD, standard deviation; PCL-R, Psychopathy Checklist–Revised; IED, Intra/Extra-Dimensional Shift; SWM, Spatial Working Memory; SST, Stop-Signal Task; RT, reaction time; SOC, Stockings of Cambridge; MITT, mean initial thinking time*.

**Figure 1:**
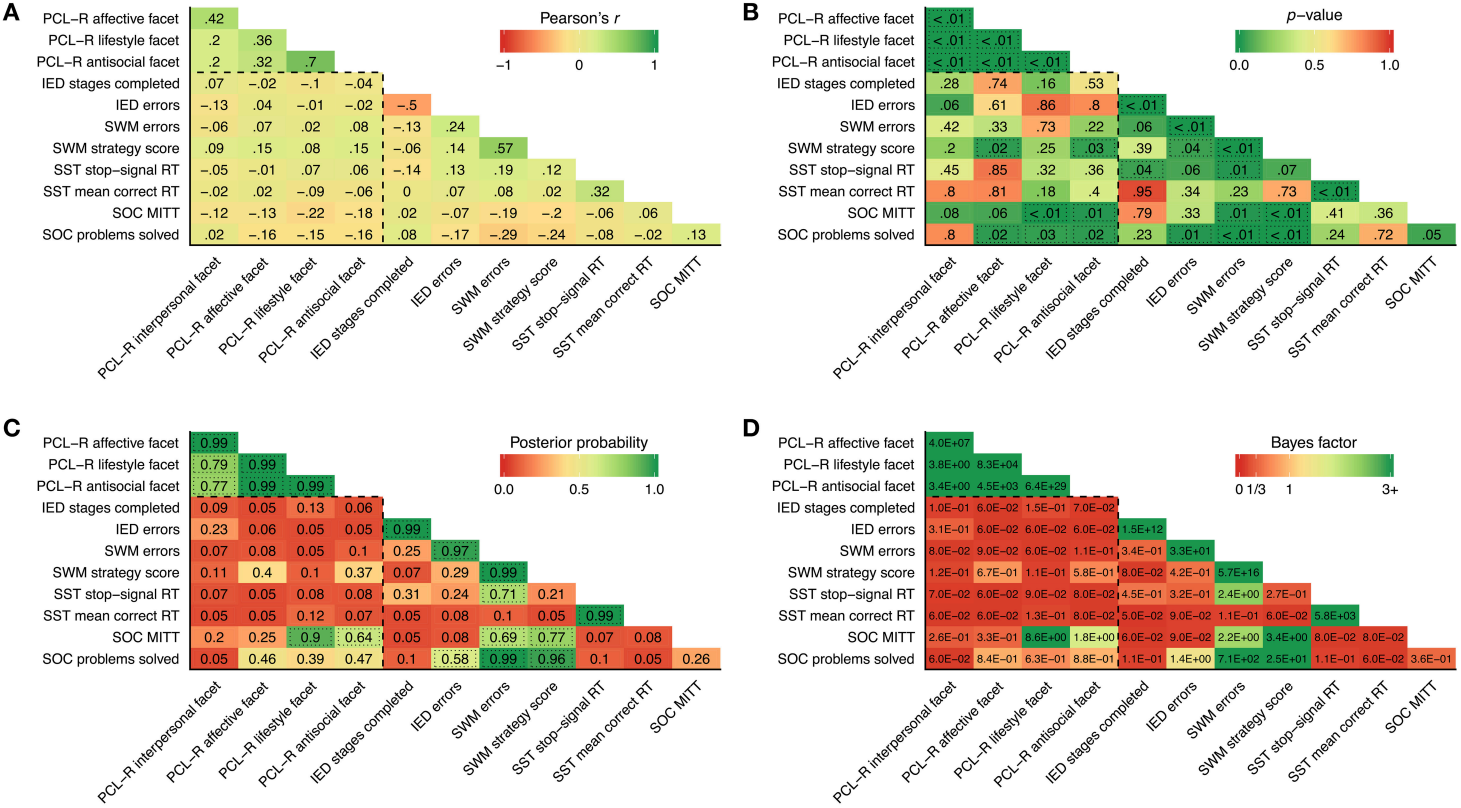
**(A–D)** Zero-order correlations (Pearson's *r*), *p*-values, posterior probabilities and Bayes factors. The dashed box contains the primary study variables. Dotted lines indicate statistical significance at *p* < 0.05 **(B)** or a posterior probability > 0.50 **(C)**. PCL-R, Psychopathy Checklist-Revised; IED, Intra/Extra- Dimensional Shift; SWM, Spatial Working Memory; SST, Stop-Signal Task; RT, reaction time; SOC, Stockings of Cambridge; MITT, Mean initial thinking time.

Lifestyle psychopathic traits were significantly and negatively associated with a lower mean initial thinking time (Figures 1A,B), corroborated by a high posterior probability (Figure 1C) and a Bayes factor indicating substantial evidence in favor of the observed correlation (Figure 1D). Similarly, antisocial psychopathic traits were significantly associated with a lower mean initial thinking time (Figures 1A,B), although the posterior probability was lower (Figure 1C), and the Bayes factor indicated anecdotal evidence in favor of the observed correlation (Figure 1D). Affective, lifestyle and antisocial psychopathic traits were significantly and negatively associated with the number of SOC problems solved (Figures 1A,B), but posterior probabilities were all < 0.50 and the Bayes factors indicated anecdotal evidence *against* the observed correlations (Figures 1C,D). Likewise, affective and antisocial psychopathic traits were significantly and positively associated with SWM strategy score (Figure 1A; also note that a high strategy score indicates poor use of strategy), but the posterior probabilities were again < 0.50, and Bayes factors indicated anecdotal evidence against the observed correlations (Figures 1C,D).

## Discussion

The present study investigated associations between four different EFs (cognitive flexibility, working memory, inhibition, planning, and problem-solving ability) and four different psychopathic traits (interpersonal, affective, lifestyle, antisocial) using zero-order correlations and a combination of classical and Bayesian statistical methods in a well-described, nationally representative cohort of young Swedish male violent offenders. Although several significant associations were observed, the subsequent Bayesian analysis indicated evidence in favor only of associations between antisocial and lifestyle psychopathic traits and lower mean initial thinking time in the SOC task. Overall, the observed effects ranged from small to medium (66).

We suggest that the association between lower mean initial thinking time and higher degrees of lifestyle and antisocial psychopathic traits could be interpreted as an impulsive approach to planning and problem-solving. Previously, (45) found that the lifestyle and antisocial facets of the PCL-R were associated with the “lack of future planning” and “acting without thinking” domains of the Barratt Impulsiveness Scale. Thus, it seems possible that in offenders high in lifestyle and antisocial psychopathic traits, behavioral manifestations of lack of planning and acting without thinking are cognitively manifested in lower mean initial thinking times. Our findings are also in line with Baskin-Sommers et al. (28), who observed a negative correlation between the impulsive-antisocial factor (i.e., factor 2) of the PCL-R and a scale score from the Tower test of Delis-Kaplan Executive Function System, which is conceptually similar to the SOC task. Overall, ours and previous results are reminiscent of early descriptions of psychopathic individuals as highly impulsive with uncontrollable desires (67) and in line with characterizations of “secondary” psychopathy (30), at least in terms of planning and problem-solving. However, some discrepancies remain. For instance, Bagshaw et al. (27), using the two-factor approach to PCL-R, found that both the interpersonal-affective and the impulsive-antisocial factors were negatively associated with planning time. They also observed greater effects from the interpersonal-affective factor, which the authors suggested may stem from difficulties in automatically switching attention when engaged in goal-directed behavior [e.g., (68)]. Our current study, however, found no significant associations between interpersonal psychopathic traits and either EF measure, indicating a need for further research to delineate the relationship between planning and problem-solving and psychopathic traits. A note of caution is also warranted as we did not include measures on subsequent thinking times and thus could not examine whether the observed lower mean initial thinking time was merely an initial impulsivity or a primary impulsive way of solving problems. Furthermore, it should be noted that a previous study adopting the broader categorical construct of psychopathy found no general impairment in planning and problem-solving, and no difference in planning time, between psychopathic individuals (PCL-R ≥ 25) and a control group (PCL-R ≤ 15) (69). A possible conclusion is that a dimensional, trait-level approach is valuable in research on psychopathy and EFs.

Directions for future research may be further uncovered by focusing on the underlying mechanisms of planning and problem-solving. It appears that while planning and problem-solving is a complex task engaging a wide range of neural regions, the dorsolateral prefrontal cortex (DLPFC) is most robustly activated (70–73). Specifically, left DLPFC activity increases under increased task load (71, 72), and left DLPFC impairments have been linked to antisocial behavior, impulsivity and behavioral disinhibition (74). Since we used measures for the highest task load possible in the current study (a five-move problem), one possibility, albeit speculative, is that the lower mean initial thinking times we observed reflects impulsivity stemming from left DLPFC impairments. Recent evidence of dysfunctional inhibitory neurotransmission in the left DLPFC in psychopathic individuals (75) lends credence to this suggestion, with evidence of increased right DLPFC activity associated with higher lifestyle and antisocial facet scores during a moral decision-making task further supporting the idea of DLPFC activity being related to specific psychopathic traits (76). Still, a wealth of research has suggested that DLPFC function might be preserved in psychopathic individuals (27, 69, 77, 78).

Another region implicated in psychopathy (79), and in antisocial behavior more broadly (74), is the orbitofrontal cortex (OFC). A previous study found that psychopathic offenders exhibited higher levels of behavioral disinhibition than non-psychopathic offenders on tasks tapping OFC function (78), and subsequent research has demonstrated that the OFC is activated during response inhibition (80). The observed impulsive behavior in patients with OFC lesions has been suggested to reflect a desire for immediate reward despite negative consequences (81). It is possible that a desire for immediate reward could manifest cognitively as lower mean initial thinking time during planning and problem-solving, in an effort to obtain the “reward” (i.e., solving the problem at hand) quickly. However, both the interpersonal-affective and impulsive-antisocial aspects of psychopathy have been linked to OFC deficits (79), while only lifestyle and antisocial psychopathic traits were associated with quicker responses in the planning and problem-solving task in the current study.

Interestingly, one aspect of spatial working memory—less efficient strategic thinking—has also been interpreted as reflecting impairments in planning and problem-solving (58). Although less efficient strategic thinking is, like planning and problem-solving, indicative of DLPFC impairments, research has suggested that working memory may not be localized to a single region but appears instead to be the result of a distributed functional network between the prefrontal cortex and the rest of the brain (82). Conceptually, planning and problem-solving is a higher order EF, likely dependent on both working memory, response inhibition, and cognitive flexibility (1). Although reduced strategic thinking was significantly associated with increased antisocial psychopathic traits, in line with previous findings (25), the finding was not corroborated by Bayesian analysis. Similarly, we also observed significant associations between increased affective traits and reduced strategic thinking, as well as fewer problems solved, in line with the suggestion that some EFs may be uniquely to the affective traits of psychopathy, but not to psychopathy as a uniform construct (28). However, neither of these results were corroborated by the Bayesian analysis, and should thus be carefully interpreted.

Finally, at least one previous study has found that increased interpersonal psychopathic traits were related to committing fewer errors in a working memory task (26). We could not replicate this finding, as we found no significant associations between interpersonal psychopathic traits and measures of spatial working memory. Overall, the relative lack of studies investigating working memory in relation to psychopathic traits, especially in correctional settings, unfortunately renders our results difficult to interpret in a wider context.

We could not replicate the many previous findings of deficit response inhibition related to impulsive and antisocial psychopathic traits (45, 48, 83–85), as neither measure from the SST showed a significant association with any psychopathic trait in the current study. A possible explanation is related to our choice of inhibition task. If participants achieve either too low (≤ 40%) or too high (≥ 60%) levels of inhibition in the SST task, which might occur if participants are distracted or otherwise perform inconsistently, the assumptions of the model are violated and no measures are available (86). The SST was placed last in the test battery, which may explain the considerable attrition in our data, with 53 participants failing to achieve proper levels of inhibition. Thus, the SST may be less suited in populations where either high (related to impulsive-antisocial psychopathic traits) or low (tentatively, related to interpersonal-affective psychopathic traits) levels of disinhibition are expected. Since previous studies of response inhibition in relation to psychopathic traits have employed other tasks, including variants of the Go/NoGo task (22, 23, 48), the Controlled Oral Word Association Test (85), the Flanker task (85), and the GoStop task (84), we recommend researchers to keep this in mind when designing future studies.

No significant associations between psychopathic traits and measures of cognitive flexibility were found in the current study. Impairments in cognitive flexibility has been observed in both violent and non-violent offenders (55, 87) as well as in offenders with antisocial personality disorder (56), and thus, at first glance, would seem related to the impulsive-antisocial traits of psychopathy. Still, we and others (44, 88) have failed to find such an association. The WCST, upon which the IED test used in the current study is based, is a complex task, requiring both working memory, inhibition, attention, error detection and conflict resolution, and along its different stages activates a large bilateral frontoparietal network (89–91). It is possible that the measures used in the current study were too broad for any significant relationships to be detected. For instance, the OFC is primarily activated during the reversal learning stage, and the anterior cingulate cortex—also suggested to be dysfunctional in psychopathy—is thought to be involved in error detection and conflict monitoring (89, 92, 93). Using more detailed measures of the different stages of the WCST and related tasks might provide further insight into the relationship between cognitive flexibility and psychopathic traits. Furthermore, it should be mentioned that there have been reports of positive associations between cognitive flexibility and the interpersonal-affective traits of psychopathy (44, 83, 94), which were not found in the current study. The positive association between interpersonal psychopathic traits and cognitive flexibility remains uncertain, and it seems that no firm conclusions can be drawn (47), indicating a need for further research.

Finally, we must mention our choice of EFs and how they were assessed. The EFs that were available in this study may be described as primarily “cool” EFs, meaning they have little emotional or contextual input. In contrast, so-called “hot” EFs involve components of motivation and affect and are more sensitive to ventromedial prefrontal cortex (vmPFC), OFC, and amygdala function than cool EFs (95, 96), which is important in light of recent research demonstrating that the neural correlates of different psychopathic traits are clearly separable (97–99). For instance, the impulsive lifestyle traits of psychopathy have been linked to impairments in the vmPFC, as well as to increased amygdala activity in response to angry facial expressions (100, 101), which might explain why these traits are also associated with reactive aggression (42). Reduced functional connectivity between the vmPFC and amygdala as well as the medial parietal cortex has been observed in psychopathic individuals (102), and reduced anterior cingulate cortex activity has been associated with higher interpersonal and affective psychopathic traits in tasks involving responses to pain stimuli (93). Thus, several aspects of the psychopathic trait dimensions may be indicative of impairments in neural regions not captured by cool EF tasks, and future studies would benefit from including tasks that tap hot EFs. In addition, while the CANTAB is a standardized battery, it is likely that tasks designed to tap a specific EF also engages other EFs, diluting the measurements, especially in higher-order EFs such as planning and problem-solving. Future studies may increase the purity of EF measure by incorporating several tasks designed to tap the same EF (3).

### Strengths and limitations

The study has some notable strengths in the large, nationally representative sample of incarcerated young violent offenders, the combination of classical and Bayesian statistical methods used, the open science practices followed, the trait-level approach to psychopathy, and the inclusion of several EF measures. The study also has several limitations that must be mentioned. The choice of EF tasks, while in line with previous literature, were selected due to the focus on neurodevelopmental disorders, and not primarily psychopathy, in the DAABS study. We also opted for an approach where the effects of each EF were investigated separately instead of using a global EF measure, which might have rendered more statistical power. Still, using a global EF makes it difficult to disentangle possible separate effects of different EFs. Another limitation is the high attrition rate in the SST task, which might have affected our results. We chose to omit participants rather than imputing the data, since we suspected non-random attrition. It should also be noted that participants scored relatively low on the interpersonal facet, compared to previous studies (26, 44, 45), and in general participants demonstrated lower PCL-R total scores than what could be expected in a sample of violent offenders. The low PCL-R scores might be due to the young age of the offenders in the current study (18–25 years), where perhaps many not yet had developed the full-blown characteristics assessed by the PCL-R. Also, cultural differences noted in previous research might contribute to the lower PCL-R scores (103). With this in mind, our results may not be generalizable outside of young male offender populations in Scandinavian or European settings.

### Summary and directions for future research

In summary, we report findings from a well-described, large and nationally representative sample of incarcerated young violent male offenders. Our results suggest that reduced initial thinking time in planning and problem-solving may be important cognitive markers for impulsive and antisocial psychopathic traits. We also tentatively propose that impairments in the left DLPFC and the OFC might be the neurobiological underpinnings of these observations. The results from this study are in line with some previous research suggesting that that specific EFs are related to specific psychopathic traits, albeit to varying degrees with small to medium effect sizes. Several discrepancies still remain, and further research is necessary. We recommend that future studies incorporate planning and problem-solving tasks in conjunction with functional neuroimaging techniques to further delineate the relationship between planning and problem-solving, DLPFC and OFC activity, and psychopathic traits. Overall, continued research would benefit from including several measures of the same EF, and also from including hot EFs.

## Author contributions

CD, MW, and BH conceived and designed the study. EB, MW, and BH obtained the data. CD drafted the initial manuscript with contributions from PA and MW. CD did all the analyses. Finally, all the authors critically revised the manuscript and approved the final version.

### Conflict of interest statement

The authors declare that the research was conducted in the absence of any commercial or financial relationships that could be construed as a potential conflict of interest.
